# Autoantibodies against Angiotensin-converting enzyme 2 and immune molecules are associated with COVID-19 disease severity

**DOI:** 10.21203/rs.3.rs-3304083/v1

**Published:** 2023-09-29

**Authors:** Todd Bradley, Eric Geanes, Rebecca McLennan, Cas LeMaster

**Affiliations:** Children’s Mercy Kansas; Children’s Mercy Hospital; Children’s Mercy Research Institute; Children’s Mercy Kansas City

**Keywords:** SARS-CoV-2, COVID-19, Antibody response, Vaccine, Autoantibodies

## Abstract

Increased inflammation caused by SARS-CoV-2 infection can lead to severe coronavirus disease 2019 (COVID-19) and long-term disease manifestations referred to as post-acute sequalae of COVID (PASC). The mechanisms of this variable long-term immune activation are poorly defined. Autoantibodies targeting immune factors such as cytokines, as well as the viral host cell receptor, angiotensin-converting enzyme 2 (ACE2), have been observed after SARS-CoV-2 infection. Autoantibodies to immune factors and ACE2 could interfere with normal immune regulation and lead to increased inflammation, severe COVID-19, and long-term complications. Here, we deeply pro led the features of ACE2, cytokine, and chemokine autoantibodies in samples from patients recovering from severe COVID-19. We identified epitopes in the catalytic domain of ACE2 targeted by these antibodies, that could inhibit ACE2 function. Levels of autoantibodies targeting ACE2 and other immune factors could serve as determinants of COVID-19 disease severity, and represent a natural immunoregulatory mechanism in response to viral infection.

The exact causes of severe COVID-19 or the long-lasting effects of SARS-CoV-2 infection, referred to as post-acute sequalae of COVID (PASC), are not yet well defined. One possible contributing mechanism could be the persistent overactivation of the immune system, driving inflammation and cellular injury disrupting host tissue homeostasis^1–3^. While infiammatory signaling is critical for viral clearance, heightened or prolonged inflammation could lead to increased tissue damage and adverse disease outcomes.

Acute cases of COVID-19 and associated disease outcomes have been shown to correlate with elevated expression levels of proinfiammatory cytokines and chemokines^4,5^. The enzyme angiotensin-converting enzyme 2 (ACE2) is not only the host viral receptor for SARS-CoV-2, but also plays a key role in the renin angiotensin system that can regulate systemic and local inflammation^6–8^. ACE2 levels have been shown to be inversely correlated with markers of inflammation, and mice with genetic knock-out of ACE2 present a hyperinflammation phenotype^6–9^. It has been reported that SARS-CoV-2 infection can directly alter ACE2 levels, leading to increased inflammation^10^. In addition to altering ACE2 levels and elevated levels of inflammation, individuals with prior SARS-CoV-2 infections produce autoantibodies targeting ACE2, type I interferons, and other immune molecules. These autoantibodies have been associated with more severe COVID-19 disease outcomes^11–20^. Interestingly, increased autoantibodies to cytokines or other autoantigens have also been observed in other respiratory infections and critical illnesses involving inflammation^21^. Thus, the generation of autoantibodies to proinfiammatory immune molecules, including ACE2, could represent a common immunoregulatory mechanism for controlling inflammation. In the case of COVID-19, investigating the levels and activity of ACE2, as well as the creation of autoantibodies, could provide insight into the extent of infiammatory responses and disease severity outcomes. Utilizing these pro les may provide a more accurate understanding of how to treat and predict COVID-19 and other respiratory disease severity prior to infection.

There have been reports that both IgG and IgM isotypes of ACE2 autoantibodies are associated with COVID-19 disease severity^13,22^. However, characterization of the features and functions of ACE2 autoantibodies and the impact on COVID-19 disease outcomes within the same cohort have not yet been described. Here, we obtained plasma samples from healthy individuals with no prior SARS-CoV-2 infection (n=38), and from individuals 30–60 days after mild (n=33; defined as no hospitalization) or severe (n=40; defined as requiring hospitalization) COVID-19 disease (**Supplementary Table 1**). We measured the levels of antibodies that targeted ACE2 using immunoglobulin (Ig) isotype-specific ELISAs. First, we used plasma samples from healthy individuals and determined individuals that were considered positive for ACE2 autoantibodies using a positivity cut-off of twice the background reading for each Ig isotype (**Supplementary Figure 1**). The highest overall levels of ACE2 autoantibodies were observed for IgA isotype, followed by IgM and IgG. We found 12 IgG, 16 IgA, and 17 IgM ACE2 autoantibody positive individuals of the 35 healthy individuals tested: indicating the presence of preexisting anti-ACE2 antibodies within healthy individuals (**Supplementary Figure 1**). Next, we compared the levels observed in the healthy individuals to those with mild or severe COVID-19. There were no significant differences in the levels of ACE2 autoantibodies of any isotype when comparing healthy individuals to individuals that had recovered from mild COVID-19 (Figure 1A–C). However, individuals with severe COVID-19 had significantly higher levels of ACE2 autoantibodies compared to healthy individuals for all three ACE2 Ig isotypes (IgG, IgA and IgM) (Figure 1A–C). These data demonstrated that ACE2 autoantibodies for all Ig subclasses could be detected, and individuals hospitalized with severe COVID-19 had higher levels of ACE2 autoantibodies after recovery compared to those that were healthy or had mild disease.

We next determined if COVID-19 vaccination altered the levels of ACE2 autoantibodies in the healthy individuals after two doses of the Comirnaty^®^ mRNA COVID-19 vaccine. After vaccination, there were no significant differences in ACE2 IgA or IgM autoantibody levels (Figure 1E, F). There was a statistically significant (P=0.002; Wilcoxon-paired test) increase in the IgG levels, but the magnitude of the change was very small (1.2 fold-change of the median) and the 95% CI of the baseline ranged from 0.06 to 0.10 and after vaccination ranged 0.08 to 0.19, indicating only a small increase in magnitude (Figure 1D). Thus, vaccination with a COVID-19 mRNA vaccine had little effect on the magnitude of ACE2 autoantibodies in healthy individuals.

Following the observation of increased ACE2 autoantibodies associated with severe COVID-19 disease, we determined whether the presence of ACE2 autoantibodies affected the enzymatic function of the ACE2 protein. We used an in vitro ACE2 functional enzymatic assay to measure ACE2 enzymatic activity by incubating plasma with enzymatically active recombinant ACE2 protein prior to the addition of an ACE2 specific uorogenic substrate and comparing the resulting fluorescence to unblocked positive control ACE2. We found that plasma from individuals with severe COVID-19 significantly decreased ACE2 enzymatic activity compared to healthy individuals not previously infected with SARS-CoV-2 (P=0.0035; Wilcoxon-Mann-Whitney; Figure 2). This indicated that peripheral blood isolated from individuals with prior severe COVID-19 infection had greater inhibition of ACE2 enzymatic activity, in addition to increased titers of ACE2 autoantibodies compared to healthy individuals.

To map the high-resolution antibody epitopes targeted by the ACE2 IgG autoantibodies in individuals with severe COVID-19, we utilized a peptide microarray that spanned the full-length ACE2 protein that consisted of a peptide library of 15 amino acids that overlapped by 11 amino acids (199 total peptides). We selected plasma from 20 individuals with severe COVID-19 containing the highest levels of ACE2 IgG autoantibodies detected in our ELISA assays to epitope map using these peptide arrays (**Supplementary Table 2**). To visualize results, we used z-scores that were computed based on the individual peptide fluorescence intensities for each individual across all the peptides that spanned ACE2 displayed as a heatmap. ACE2 consists of two major domains, a zinc metallopeptidase domain (blue) and a collectrin-like domain (yellow) (Figure 3A). The motifs required for substrate binding, enzymatic activity, and interaction with the SARS-CoV-2 spike protein (red) are contained in the zinc metallopeptidase domain^23–26^. The collectrin-like domain contains cleavage sites for ADAM18, TMPRSS11D and TMPRSS2 peptidases (black) and several linear binding motifs for LIR, PDZ-binding, PTB and endocytic sorting signal motifs (green) that are important for trafficking and autophagy^27–29^ (Figure 3A). Antibody binding to peptides across the entirety of the ACE2 protein occurred throughout the array of individuals, however there were common epitopes targeted by multiple samples. To quantify this, we calculated the median z-scores for each peptide across all 20 individuals and defined a z-score of ≥1 (representing 1 standard deviation above the median) as high binding (Figure 3B (gray), **3C**). Seven regions met this criterion, with only two regions containing more than one peptide with high binding (Figure 3B, **Supplementary Table 3**). None of the high binding regions overlapped with the regions of ACE2 previously shown to be important for interacting with SARS-CoV-2^23,24,27,28^. One of the regions with the highest binding did involve residues important for substrate binding (Figure 3B, C). Mutation of the arginine at residue 273 has been shown to block substrate binding and abolished enzymatic activity when mutated^25^. This data showed that ACE2 autoantibodies had common linear epitopes across many individuals and recognize regions important for ACE2 enzymatic activity.

In addition to autoantibodies targeting ACE2, autoantibodies to cytokines, chemokines, and other immune molecules have also been associated with COVID-19 disease severity^14,15,17–19^. Therefore, we utilized a bead-based fluorescence Luminex assay to determine the levels of autoantibodies against 23 cytokines, chemokines and immune molecules for IgG, IgA and IgM isotypes. We found high levels of autoantibodies were present to certain cytokines in the healthy individuals, but most analytes displayed large levels of heterogeneity (Figure 4A). IgG autoantibodies against IL-4, IL-17F, IL-17A, IFNω and IFNγ had the highest median levels in the healthy group (Figure 4A). IgM levels were significantly higher than most analytes compared with IgG, with the exception of IL-4, IL-15, and IL-22 where IgG exhibited significantly higher levels (Figure 4B). Conversely, IgA levels to all the analytes, except osteopontin, were significantly lower than IgG levels (Figure 4C). Thus, autoantibodies to cytokines, chemokines, and other immune factors could be detected in healthy individuals at different magnitudes, depending on the individual and analyte. Moreover, there were distinct patterns based on Ig isotype. This could indicate a natural immunoregulation system that involves antagonism of cytokines and other immune molecules through antibody-mediated mechanisms, that has individual variation, even in healthy populations.

Next, we compared the patterns of autoantibodies in the severe COVID-19 individuals to the healthy controls. For the IgG autoantibodies, the severe COVID-19 group trended for higher levels to many of the analytes tested and had statistically significant higher levels to both IL-3 and IFNα2 cytokines (p≤0.05; Wilcoxon-Mann-Whitney; Figure 5A, **Supplementary Figure 2**). Similarly, the severe COVID-19 group had higher levels of IgA autoantibodies to most cytokines with 18 of the 23 analytes significantly higher in the severe COVID-19 group compared to healthy controls (p≤0.05; Wilcoxon-Mann-Whitney; Figure 5B, **Supplementary Figure 2**). There were no significant differences in IgM autoantibody levels between severe COVID-19 and healthy groups (**Supplementary Figure 2**). Subsequently, we determined if any cytokine autoantibody levels were correlated with one another in the IgG isotype. Interestingly, there were groups of correlating analytes in the severe COVID-19 group that were not detected in the healthy group (Figure 5C). In the severe COVID-19 group, IL-1α, IFNα2, TNFα, osteopontin and IFNβ were one of the largest groups of analytes that had correlated expression levels (Figure 5C). These data identified that autoantibodies to cytokines, chemokines, and other immune factors were present in healthy individuals at varying degrees of magnitude and are dependent on Ig isotype. Moreover, individuals with severe COVID-19 had significantly higher levels of autoantibodies to these isotypes for both IgG and IgA, but not IgM.

In this study we showed that autoantibodies of all major Ig subclasses (IgG, IgA, IgM) targeting the SARS-CoV-2 viral host cell receptor ACE2 were present in the blood, and the levels were associated with COVID-19 disease severity. We found that individuals hospitalized with COVID-19 had higher ACE2 autoantibody levels of all three Ig isotypes. This correlation of ACE2 antibodies with disease severity is consistent with previous studies, but this is the first study demonstrating increased ACE2 antibody levels in IgG, IgA and IgM based on disease severity^11,13,15,16^. Moreover, we showed that individuals with higher levels of ACE2 autoantibodies could block the enzymatic function of ACE2, indicating that these antibodies could impact ACE2 physiological function. Indeed, SARS-CoV-2 infection has been associated with increased ACE2 shedding from cell membranes and increased plasma ACE2 catalytic activity^11,30^. Apart from its role as the SAR-CoV-2 receptor, membrane bound ACE2 typically regulates blood pressure, wound healing, and inflammation via the renin angiotensin system^6–8^. By disrupting the balance of ACE2 through decreasing membrane bound ACE2 and increasing autoantibodies binding to enzymatically active epitopes, enzymatic activity would decrease, and functions dependent on ACE2 regulation, such as inflammation, would be impacted and unregulated. Consistent with this observation, when ACE2 was knocked out in mice, proinfiammatory cytokines were induced and inflammation was increased in response to minor stress^9^.

We used peptide microarray to map the high-resolution peptide binding of the ACE2 autoantibodies to the ACE2 protein. We found unique and common epitopes that were targeted by ACE2 autoantibodies in individuals with severe COVID-19. Importantly, some of these binding sites were located within enzymatically active domains and could have an impact ACE2 function. Future studies could determine if antibodies targeting these epitopes could block ACE2 activity and identify how B cells making these autoantibodies develop. Additionally, these antibody epitopes could be further explored as peptide biomarkers of COVID-19 disease severity.

Lastly, we found the presence of IgG, IgA, and IgM autoantibodies to a multitude cytokines, chemokines, and other immune factors within healthy individuals, and identified autoantibodies to immune factors that were significantly higher in individuals with severe COVID-19. Even though autoantibodies exist at a basal level within healthy individuals, this study provides evidence that elevation of these autoantibodies, including ACE2 autoantibodies, is related to severity of disease with exposure to SARS-CoV-2. This finding is consistent with previously published data, with reports of as much as 60% of hospitalized COVID-19 patients presenting with higher autoantibodies to certain antigens^12,14–16^. Patients with severe COVID-19 produced higher amounts of circulating neutrophils, and increased neutrophil activation, when compared to patients suffering from milder forms of COVID-19^2,20^. Furthermore, patients infected with SARS-CoV-2 showed elevated levels of cytokines, produced by neutrophils (IL-10, IL-8 and IL-6), which is consistent with our findings^2^. Wang et al. performed a large profiling for autoantibodies against 2,770 proteins on a cohort of 194 individuals and discovered widespread autoantibody increases, with a striking enrichment in autoantibodies targeting interferons in patients with the most severe cases of COVID-19^15^. These researchers further demonstrated that pretreating transgenically expressed human ACE2 mice with neutralizing antibodies against interferon alpha/beta receptor prior to SARS-CoV-2 infection led to increased disease severity and decreased survival^15^. Finally, over 10% of the patients with COVID-19 (from a large cohort of 987) had neutralizing IgG autoantibodies to type 1 interferons that could functionally neutralize type 1 interferons abilities to block SARS-CoV-2 infection in vitro^14^. Whether these autoantibodies target COVID-19 specific proteins or whether their upregulation is a more global response seen in multiple in severe illnesses is still debatable^4,21,31^. Using a short longitudinal study, it was determined that a subset of autoantibodies were triggered specifically by SARS-COV-2 infection, but these patients were only followed for 7 days^12^. Interestingly, a recent study reported that 12 months after infection, patients with mild COVID-19 had higher autoantibody levels to cytokines such as CCL21, CXCL13 and CXCL16, compared to long COVID-19 patients^31^. This finding is inconsistent with other reports, including a report examining immunological dysfunction 8 months after SARS-CoV-2 infection^4^, but may suggest that certain autoantibodies could positively influence long term disease outcomes^31^. Whether the same autoantibodies are elevated during brief or prolonged time frames after infection is yet to be determined. Additionally, whether the same autoantibodies are triggered by other respiratory infections for example is still under investigation. A recent study examined IgG autoantibodies in 267 patients suffering from non-SARS-CoV-2 acute illnesses and discovered that autoantibodies are indeed upregulated in response to a wide variation of diseases and illnesses^21^. Specifically anti-cytokine antibodies, such as TNFα, IL-2, IL-17A and IFNα interferons, were found in all acute illnesses examined, with significantly higher levels in infected versus healthy individuals^21^.

It is currently unclear whether all of the autoantibodies expressed at higher levels after COVID-19 disease are truly elevated in response to infection or whether some patients are predisposed to higher levels prior to infection, which in turn contribute to severe symptoms^32,33^. Whether autoantibodies are the cause or the result of severe COVID-19, our study and others suggest that these autoantibodies may be used as predictive markers of disease severity in COVID-19 and other infections. Future work understanding how these autoantibodies develop and the mechanisms to block or enhance inflammation could uncover novel therapeutic approaches to controlling inflammation.

Our data demonstrated that SARS-CoV-2 infection can increase autoantibodies and show a correlation between elevated autoantibodies and COVID-19 disease severity. However, one limitation is our study size, demographic makeup and longitudinal follow-up. Moreover, determining if the autoantibodies truly contribute to immune regulation in vivo will be critical for determining the functional impact of this observation. Larger, longitudinal studies of diverse individuals will be required to fully characterize the fate and function of these autoantibodies to ACE2 and cytokines.

## Methods

### Human Subjects

Healthcare workers from our children’s hospital were enrolled prior to the administration of the Comirnaty^®^ mRNA COVID-19 vaccine (Pfizer-BioNTech, New York, NY, USA). Plasma from peripheral blood was collected before vaccination as a baseline (week 0) and after second immunization (week 7) from individuals with no known history of infection (n=38) or with PCR laboratory-confirmed SARS-CoV-2 infection and no hospitalization (n=33). Sample population consisted of mostly adult middle aged, white, females who did not identify as Hispanic or Latino (**Supplementary Table 1**). Comirnaty^®^ vaccine biospecimens were collected under a research study at Children’s Mercy Kansas City. This study was reviewed and approved by the Children’s Mercy IRB (#00001670 and #00001317). Participants self-enrolled after they had reviewed a study information letter and were given opportunity to ask questions. IRB waived written informed consent after these criteria were met.

Severe COVID-19 convalescent biospecimens, 30–60 days after infection, were obtained through Boca Biolistics, LLC (Pompano Beach, FL, USA) and were collected under a clinical study that has been reviewed by an Institutional/Independent Review Board (IRB) and/or Independent Ethics Committee (IEC) in accordance with requirements of local governing regulatory agencies including the Department of Health and Human Services (DHHS) and Food and Drug Administration (FDA) Codes of Federal Regulations, on the Protection of Human Subjects (45 CFR Part 46 and 2l CFR Part 56, respectively) (**Supplementary Table 1**). These convalescent individuals that had PCR laboratory-confirmed SARS-CoV-2 infection and were hospitalized. Serum or plasma was isolated from venous whole blood collection and stored frozen in ultra-low temperature freezers until used to perform immunoassays.

### ACE2 Enzyme-linked immunosorbent assays

ELISAs were performed using ACE2 recombinant protein (Cat# HCYTAAB-17K, Sino Biological, Wayne, PA, USA) diluted to 2 ug/mL in 0.1 M sodium bicarbonate and incubated on high-binding plates (3369, Corning Inc, Corning, NY, USA) overnight at 4 degrees. Serum or plasma was diluted to 1:50 in superblock buffer with sodium azide. Secondary antibodies were purchased from the following: Goat anti-human IgG (Cat# 109-036-098, Lot# 149163, Jackson ImmunoResearch, West Grove, PA, USA), Goat Anti-Human IgA alpha chain (Cat# ab97215, Lot# GR3373878–8, Abcam, Waltham, MA, USA), Goat Anti-Human IgM mu chain (Cat# ab97205, Lot# GR3396429–1, Abcam, Waltham, MA, USA). Secondary antibody dilutions were done in superblock buffer without sodium azide within range of manufacturer’s recommendations at: IgG 1:50,000, IgA 1:1000, and IgM 1:2000 dilution. SureBlue Reserve Microwell Substrate (95059–294, VWR, Radnor, PA, USA) was added and incubated in the dark for 15 minutes. Absorbance was measured at 450 nm immediately after 0.33 N HCl Acid Stop solution was added to the plate. Positive baseline cutoff was determined by values greater than or equal to twice the background OD450.

### ACE2 Inhibition Assay

Detection of antibodies inhibiting ACE2 activity was determined using the ACE2 Inhibitor Screening assay kit (Cat# 79923, AMSBIO, Cambridge, MA, USA). 5 uL of serum or plasma was added to 20 uL of 0.5 ng/mL ACE2 enzyme solution. 25 uL of ACE2 uorogenic substrate was added to a final volume of 50 uL. Following the manufacturers protocol, fluorescence intensity (excitation 555, emission 585) was measured for all wells after 60 minutes at room temperature. All wells were normalized to blank well fluorescence intensity. Percent ACE2 inhibition was determined by dividing test well values by the average positive control well value.

### ACE2 peptide microarray

A peptide library of 15 amino acids that overlapped by 11 amino acids (199 total peptides) that spanned the entire ACE2 protein were synthesized by JPT peptide Technologies (Berlin, Germany) using PepStar technology that covalently immobilized the peptides onto glass microarray surfaces using an optimized hydrophilic linker moiety. Full-length human and mouse IgG were co-immobilized on microarray slides as assay controls. The 20 severe COVID-19 serum samples were diluted 1:200 and incubated for 1 hour at 30 °C on multiwell microarray slides. After incubation and washing, fluorescently labeled anti-human-IgG antibody at 0.1 μg/mol was added to the wells and incubated for 1 hour. Additional control incubations with secondary antibody only (with no serum samples) were also performed in parallel on each slide to assess false positives. After washing and drying, the slide was scanned with a high-resolution laser scanner (GenePix; Molecular Devices, San Jose, CA, USA) at 635 nm to obtain fluorescence intensity pro les. Resulting images were quantified to yield a mean pixel value for each peptide. Blocking buffer was Superblock TBS T20 (Pierce International) and wash buffer was 50mM TBS-buffer including 0.1% Tween20, pH 7.2.

### Autoantibody multiplexed binding assay

To measure autoantibody levels against cytokines commonly associated with autoantibody disease, 15 cytokines were used on a bead-based multiplex assay based on the Luminex xMAP technology (Austin, TX, USA). Reagent kits with secondary antibodies specific for immunoglobulin G (IgG) were used (HCYTAAB-17K, MilliporeSigma, Burlington, MA, USA) following manufacture protocol. The kit provided a set of 15 antigen conjugated beads (BAFF/Blys, G-CSF, IFNβ, IFNγ, IL-1α, IL-6, IL-8, IL-10, IL-12 (p40), IL-15, IL-17A, IL-17F, IL-18, IL-22, TNFα) along with 3 positive control beads and a negative control bead set. The positive control beads were beads coated with different concentrations of IgG. The negative control beads did not have antigen conjugated to determine nonspecific binding. The 15 antigen-conjugated beads, 3 positive control beads, and 1 negative control beads were mixed and incubated with each plasma sample at a dilution of 1:100 with assay buffer. Two wells with only buffer and no plasma were used to determine background activity. PE-anti-human IgG conjugate detection antibody was utilized to determine antibody response to each cytokine. We utilized the Luminex analyzer (MAGPIX) and Luminex xPONENT acquisition software to acquire and analyze data. After acquisition net MFI was calculated by subtracting background MFI (no plasma).

### Statistical Analysis

The statistical analysis was performed using Graphpad Prism 9.1 (Boston, MA, USA). For multiple comparison, the statistical significance was determined with a Wilcoxon-Mann-Whitney test with two-tailed P values.

## Figures and Tables

**Figure 1:**
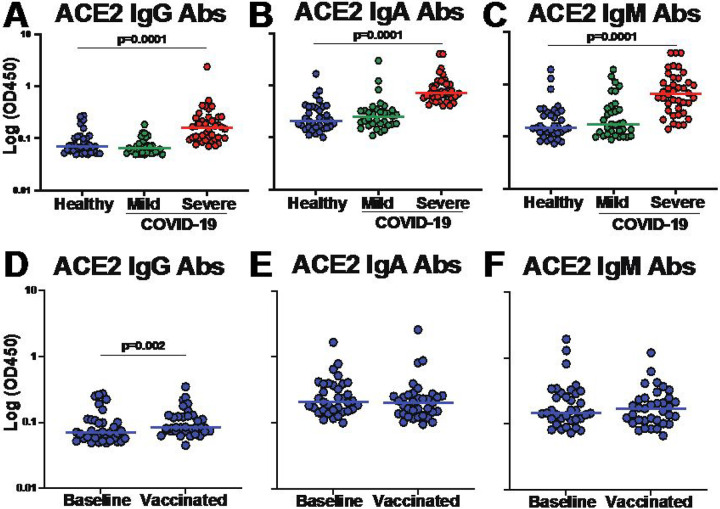
ACE2 autoantibody levels are increased in severe COVID-19. **(A-F)** Dot plot graphs of OD450 values on log axis obtained by ELISA for determining the levels of antibodies targeting ACE2 within plasma from healthy individuals (n=35, blue), individuals with mild COVID-19 (not hospitalized, n=33, green), and individuals with severe COVID-19 (hospitalized, n=40, red). Secondary detection antibodies specific for Ig isotype were utilized to identify **(A)** IgG ACE2 antibody levels **(B)** IgA antibody levels and **(C)** IgM antibody levels. **(D-F)** Dot plots showing ACE2 antibody levels in healthy individuals at baseline and then after two doses of Comirnaty COVID-19 mRNA vaccination. Ig Isotype specific ACE2 antibodies determined for **(D)**IgG **(E)** IgA and **(F)** IgM. Each dot represents a single individual and Wilcoxon–Mann–Whitney statistical test was used to determine P values.

**Figure 2:**
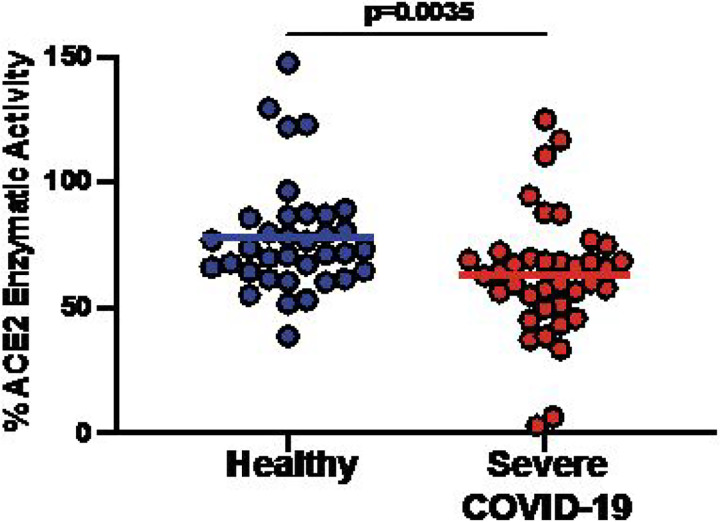
Plasma from individuals with severe COVID-19 inhibited ACE2 enzymatic function. Recombinant ACE2 was combined with plasma prior to the addition of an ACE2 specific uorogenic substrate to quantify plasma inhibition of ACE2 enzymatic activity. Dot plot of percentage of ACE2 enzymatic function detected when plasma from healthy (n=35, blue) and severe COVID-19 (n=40, red) individuals added compared to control where no inhibition (plasma) is added. Each circle represents distinct individuals. Lines represent the mean of all the samples. P value was determined with a Wilcoxon–Mann–Whitney test.

**Figure 3:**
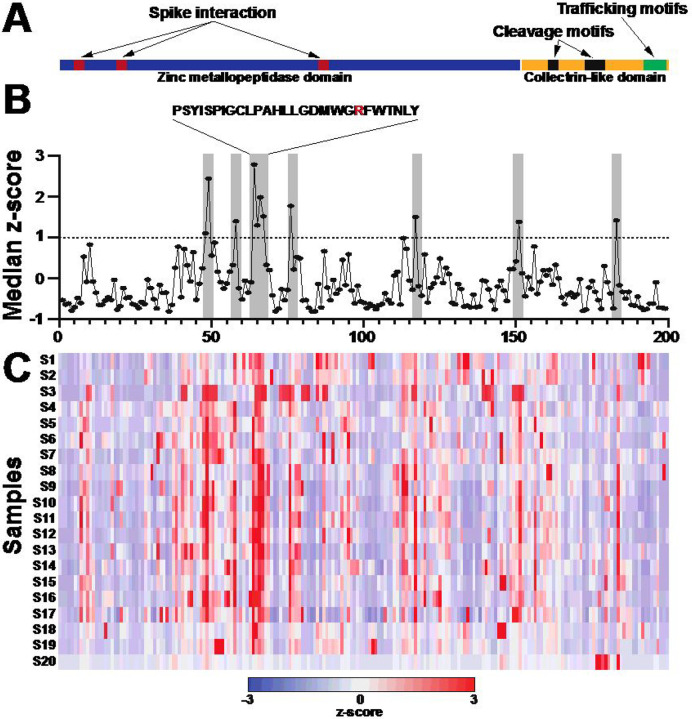
High-resolution antibody epitope mapping using ACE2 peptide microarray identified regions of targeted binding in individuals with severe COVID-19. **(A)** Schematic of ACE2 protein over peptide regions with known domains and motifs highlighted. **(B)** Line graphs of median group z-scores of ACE2 autoantibody binding to individual ACE2 peptides. Epitopes of recurrent high binding were qualified as median z-scores of ≥1 to represent 1 standard deviation above the median. Seven regions qualified as high binding (grey). (C) Heatmap of z-scores for ACE2 epitope binding for individual samples, n=20.

**Figure 4:**
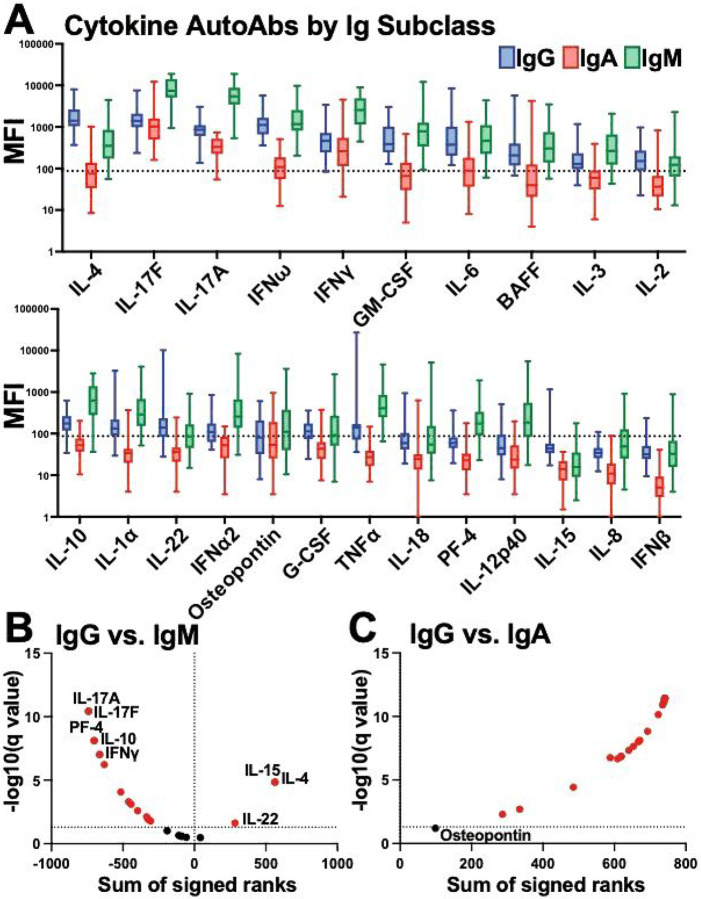
Autoantibodies for cytokines, chemokines, and other immune factors were detected for different Ig isotypes within healthy individuals. **(A)** Multiplex bead-based antibody binding assay measured the levels of autoantibodies against 23 cytokines, chemokines and immune molecules for IgG (blue), IgA (red) and IgM (green) isotypes within healthy individual plasma samples (n=38). Median Fluorescent Intensity (MFI) was calculated; background subtraction was used to remove nonspecific signal. The dashed line indicates a threshold determined by the sum of the mean and standard deviation for the negative control (i.e., beads without antigen). **(B-C)** Volcano plots showing the results of multiple Wilcoxon matched pairs test that was FDR corrected for multiple comparisons (Benjamini) that compared each analyte **(B)**IgG vs IgM expression levels or **(C)** IgG vs. IgA expression levels. Dotted lines indicate FDR<0.01.Analytes highlighted in red are significantly changed between antibody isotypes compared.

**Figure 5:**
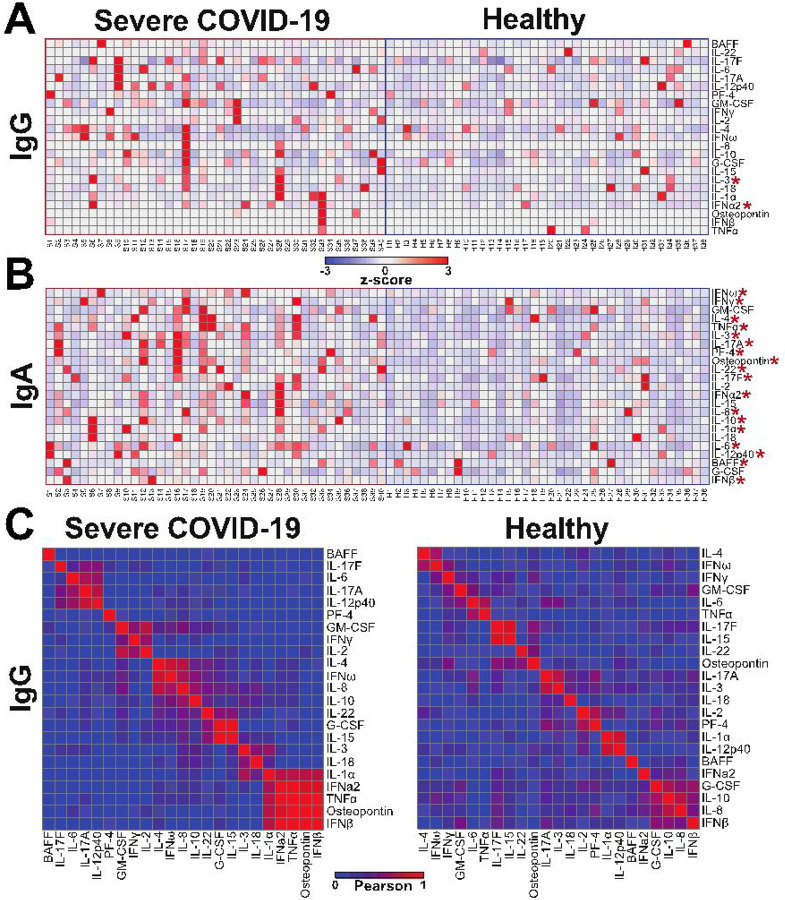
Individuals with severe COVID-19 had significantly higher autoantibodies to distinct analytes for IgG and IgA. **(A-B)**Heatmaps of row z-scores of **(A)** IgG and **(B)** IgA autoantibody levels to the 23 cytokines, chemokines and immune factors tested. Individuals with severe COVID-19 disease (left, n=40) and healthy controls (right, n=38). Analytes significantly different in individuals with severe COVID-19 compared to controls are shown with a red asterisk. *, p≤0.05; Wilcoxon-Mann-Whitney. **(C)** Pearson correlations of the IgG antibody levels between each analyte for severe COVID-19 individuals and healthy individuals.

## Data Availability

The data that support the findings of this study are available from the corresponding author, T.B., upon reasonable request.
